# Unified Theory for Flexural Strengthening of Masonry with Composites

**DOI:** 10.3390/ma12040680

**Published:** 2019-02-25

**Authors:** Giancarlo Ramaglia, Francesco Fabbrocino, Gian Piero Lignola, Andrea Prota

**Affiliations:** 1 Department of Structures for Engineering and Architecture, University of Naples Federico II, Via Claudio 21, 80125 Naples, Italy; giancarlo.ramaglia@unina.it or giancarlo.ramaglia@unipegaco.it (G.R.); aprota@unina.it (A.P.); 2 Department of Engineering, Telematic University Pegaso, Piazza Trieste e Trento, 48, 80132 Naples, Italy; francesco.fabbrocino@unipegaso.it

**Keywords:** masonry, strengthening system, inorganic matrix, dimensionless form, out-of-plane, sensitivity analysis

## Abstract

Recent calamitous events have shown the fragility of the existing masonry buildings. Many of them are heritage structures, such as churches and monumental buildings. Therefore, optimized strengthening strategies are necessary. Experimental studies performed on masonry elements strengthened with composite systems have shown the performance of these materials. However, further development is necessary to optimize the intervention strategies. In fact, due to the lack of general validity models, the design is usually based on prescriptive approaches according to manufacturers’ broad instructions, often producing systems with low efficiency and overestimations of the amount of reinforcement. In this paper a generalized approach is proposed to assess the flexural behavior of masonry sections strengthened with composites. The proposed theory has allowed performance of a sensitivity analysis assessing the impact both of the mechanical parameters of masonry and of the strengthening system. In particular, the impact of several constitutive relationships of composites (linear, bilinear, or trilinear) have been evaluated in terms of ultimate behavior of the strengthened masonry. For strengthening systems more compatible with the masonry substrate, the form of the stress–strain relationship becomes a key aspect. For such cases, the modeling of the reinforcement plays a fundamental role and the form of the relationship is strongly correlated to the type of reinforcement selected, e.g., organic versus inorganic matrix.

## 1. Introduction

In recent years, especially after recent earthquakes, enormous progress has been made in the understanding of structural behavior and the development of efficient intervention strategies on existing masonry structures [1,2]. The mitigation of the seismic risk is particularly important, because a large part of the existing heritage is made of masonry buildings (churches and monumental buildings). Recent seismic events in Italy, such as L’Aquila (2009), Emilia Romagna (2012), and Central Italy (2016), have shown, in addition to the issue of the seismic vulnerability of existing cultural heritage sites [3,4], the need to strengthen using compatible and efficient structural strengthening techniques [5,6], and sometimes also to repair previous interventions [7]. Although masonry is the oldest building material, the structural behavior of masonry structures is hard to assess [8,9]; difficulties are often due to the great heterogeneity of response that leads to conceiving the structural strengthening intervention in a cautious way [10].

In the strengthening of masonry structures, new technologies based on Fiber Reinforced Polymers (FRP)—that is, with an organic resin matrix—or Fiber Reinforced Cementitious Matrix (FRCM) with an inorganic matrix [11] have been recognized for decades (FRP in particular). Their developing progress is due to high compatibility with the masonry substrate [12,13], especially for FRCM with lime-mortar matrix in high-quality building applications preserving the historical identity of structures. Despite development in the application field, further theoretical developments are needed to fully understand the influence of the characteristics of strengthening systems on the behavior of strengthened masonry elements. To date there is no general consensus on calculation models for the design and verification of masonry elements [14] strengthened with composites. Many of the available models in the literature and in the international guidelines and codes [15,16] are not able to accurately account for the behavior of strengthening systems on the final response of strengthened masonry elements. These models provide reliable results for FRP, where the organic matrix contribution can be neglected. Conversely, peculiarities of FRCM due to matrix stiffness and strength provide non-negligible effects on ultimate behavior of strengthened masonry elements. In practical applications, in the lack of general validity models, the design is usually based on prescriptive approaches according to manufacturers’ instructions, often producing systems with low efficiency or even deleterious effects for the ductility of strengthened elements [17,18]. In over-strengthened elements, the failure is promoted in the masonry, hence there is a further risk for the conservation of the masonry heritage value.

Under seismic actions, increasing the capacity alone may not be the only solution. In fact, ductility is an essential parameter to check and control, especially for masonry structures. This paper focuses mainly on FRCM strengthening systems, in particular on the assessment of the ultimate capacity of strengthened masonry sections. The model is developed for the analysis of the ultimate behavior of strengthened masonry sections using FRCM (and FRP). The approach is applicable to both the cases of in-plane and out-of-plane bending of masonry, however the strengthening system is loaded with a strain gradient while in-plane, and it is all loaded at the same level in the out-of-plane case; in the discussion and sensitivity analyses, the results of the out-of-plane case only are discussed. In the specific case, starting from the moment-curvature M-χ diagrams, the P-M domains of the strengthened sections are obtained. The M-χ diagram is a key element in the structural design, especially in the field of strengthening and repair of the built heritage, to be able to analyze the behavior of structural elements, both from the point of view of load capacity but also of the ductility according to modern performance-based design approaches [19,20]. The calculation model makes it possible to analyze the behavior of the strengthened masonry sections, parametrically, as the characteristics of both the masonry substrate and the strengthening system changed. The calculation was based on a "Stress Block" refined approach.

The proposed calculation model allowed the development of sensitivity analyses and the obtained results have been expressed completely in a dimensionless form, independently of the geometrical and mechanical parameters of the section and of the strengthening system. This approach allowed us to obtain results that can be fully generalized, and therefore applicable to any masonry structural framework. The results presented in this paper represent the basis for the development of standardized design or verification methodologies for the analysis of the flexural behavior of masonry structures strengthened with FRCM.

## 2. Stress–Strain Behavior of Materials

### 2.1. Behaviour of Masonry

In the scientific literature, there are many stress–strain relationships to describe the mechanical behavior of masonry [21,22]. The “masonry” material has a stress–strain relationship characterized by a non-linear behavior and by strengths in compression and tension in between those of the constituent materials. In the analysis of masonry structures, it is therefore not possible to limit the analysis to the linear behavior, but it is required, necessarily, to analyze also the nonlinear behavior [23]. The limit of the linear behavior coincides usually with the beginning of the cracking of the section [24], but this phenomenon, for a material with very low tensile strength or even zero tensile strength as the masonry [25,26], already occurs at very low axial load levels compared to the axial capacity. Experimentally, it has been observed that the tensile strength is between 2% and 7% of the compressive strength [27].

**Figure 1:**
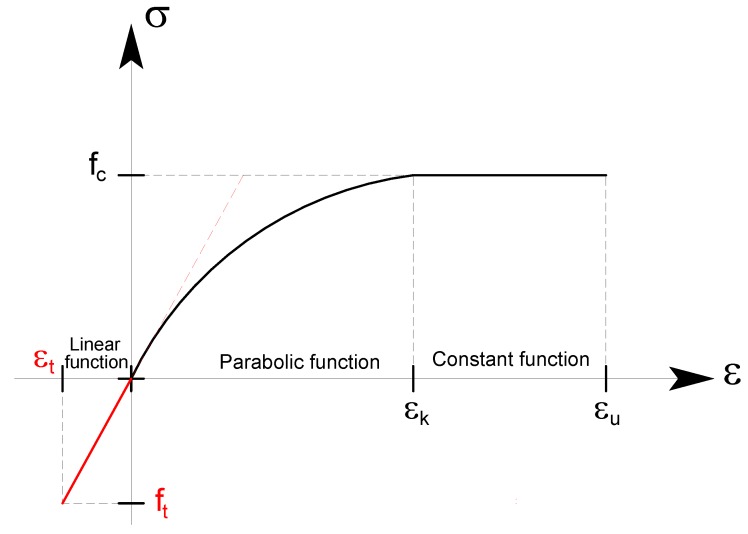
Stress–strain relationships of the masonry—compression (positive) and tension (negative).

For the present work, different stress–strain relationships in compression and tension have been used. In compression, the model suggested by the Eurocode, EC6, was used, [15] where the post-peak softening (Figure 1) is neglected, idealizing the behavior after peak as perfectly plastic.

The equation proposed by the Eurocode EC6 is verbatim reported below:

(1) $$\{\begin{array}{ll} \frac{\sigma }{f_{c}}=2\cdot \left(\frac{\varepsilon }{\varepsilon_{k}}\right)-{\left(\frac{\varepsilon }{\varepsilon_{k}}\right)}^{2} & per\;0\leq \varepsilon \leq \varepsilon_{k}\\ \frac{\sigma }{f_{c}}=1 & per\;\varepsilon_{k}\leq \varepsilon \leq \varepsilon_{u} \end{array},$$

with *ε_k_* = 0,0020 and *ε_u_* = 0,00350.

The Eurocode suggests a modulus of elasticity equal to:

(2) $$E_{em}=1000\cdot f_{c},$$

where *f_c_* is the compressive strength of the masonry. It is also the tangent at origin of the parabolic function (1).

On the other hand, in tension, the material has an elastic-brittle behavior (Figure 1), is linear, with a slope equal to *E_em_* and ultimate strain equal to the ratio between the tensile strength of the masonry and the modulus of elasticity:

(3) $$\varepsilon_{t}=-\frac{f_{t}}{E_{em}}.$$

### 2.2. Behavior of Strengthening System

In the wide industry of reinforcement systems in civil engineering, FRP certainly gained a wide dominance. Given some shortcomings in terms of durability, fire resistance, and applicability on wet surfaces, the industry developed other types of composites based on inorganic matrices, instead of organic resins, mainly consisting of mortars enriched with fibers and additives: in this case, the most common acronym is FRCM.

The FRCM, as already mentioned in the previous paragraph, is a strengthening system consisting of one or more layers of fiber grids or fabrics impregnated within an inorganic matrix, cementitious or lime based, whose ultimate properties and modulus of elasticity depend on the type of fiber and the mortar used as reinforcement and matrix, respectively. However, it is possible to provide a general stress–strain relationship able to simulate the behavior of the system as a whole, taking into account the factors that influence it most, such as shrinkage and cracking of the matrix, and slip and debonding phenomena between the reinforcement fibers and the matrix, and eventually the matrix and the substrate [28].

The stress–strain relationship of these composites is variable; basically, the fibers have a linear behavior, while the matrix provides a tension stiffening effect at the early stages [29,30]. In the case of organic matrix (FRP), the matrix contribution is negligible [31,32], hence the global stress–strain behavior can be assimilated to the behavior of the fibers (linear behavior); conversely, in the case of inorganic matrix (FRCM), the early stiffening of the mortar matrix is clear and the behavior usually has three stages [32]. The first uncracked portion is dominated by the behavior of the matrix [33] and is characterized by a modulus of elasticity, E_1_, greater than in the subsequent phases. For low strain levels, the composite is completely active and has considerable stiffness [34].

In the second phase, the modulus of elasticity, E_2_, is considerably reduced [28,29], because the tensile strength of the matrix is overcome with the consequent formation of cracks [35]. There is the transition from the uncracked phase to complete cracking, which is comparable to what happens in reinforced concrete, due to the phenomenon of tension stiffening [36,37]. The cracks in the matrix should occur before the failure of the composite, hence there should be enough fiber volume to prevent failure of fiber at matrix cracking, which is to prevent a very brittle failure; however, the transition phase of matrix cracking depends strongly on the matrix performance. In the case of a high performance matrix, there is a gradual reduction of stiffness and the failure involves the fibers with a good matrix contribution, hence with a clear bilinear behavior. If the fiber contribution reduces before failure, the stress–strain relationship is similar to the behavior of the dry fibers close to failure, with a final third showing almost linear behavior [38]. In this phase the internal fibers could slip with respect to the matrix [39], providing some ductility due to the fact that the failure of the fibers does not take place at the same time, but progressively over time [40]. The third phase ends with the achievement of the tensile failure of the fibers with a slope equal to E_3_, the value of the modulus of elasticity of the dry fibers [41]. As a lower bound case, a horizontal plateau could be considered after cracking of the matrix and before the stretching of the dry fibers, as a limit case of the low performance matrix (with a clear trilinear behavior).

In the following, the stress–strain relationships of the composite system are simulated in three ways through three types of relationships: linear behavior, bilinear behavior, and trilinear behavior. The representativeness of the behavior depends on the characteristics of the system, as remarked in various experimental studies [42,43].

#### 2.2.1. Linear Behavior

**Figure 2:**
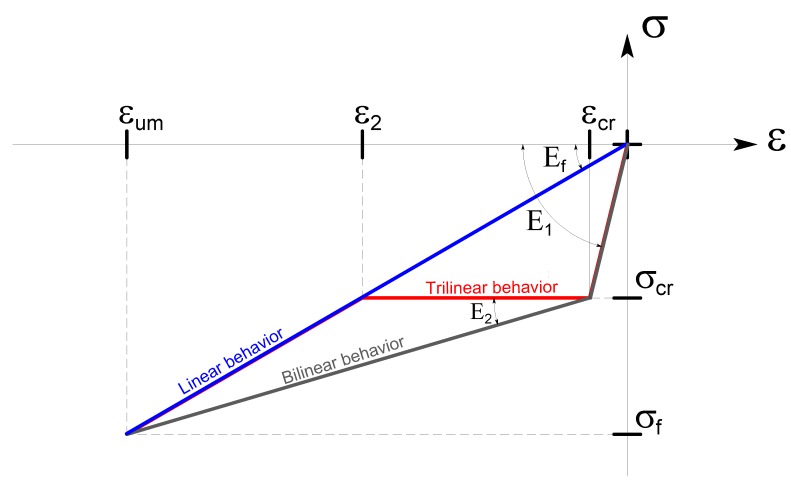
Different stress–strain relationships for composite systems (homogenized with respect to the dry fiber).

Assuming a linear behavior, the stress–strain relationship of the composite system has been simplified to a single linear relationship (Figure 2), in which the system reaches the ultimate tensile stress of the fiber with a slope equal to the modulus of elasticity of the dry fiber, *E_f_*:

(4) $$E_{f}=\frac{\sigma_{um}}{\varepsilon_{um}},$$

where:

$\sigma_{um}$ is the ultimate stress of the fiber (it can be related also to debonding stress of composite);
$\varepsilon_{um}$ is the corresponding ultimate strain of the composite.

This represents the behavior of the FRP where the matrix contribution is negligible in this aspect, or an FRCM where the matrix cracking is premature and early compared to fiber failure, hence matrix impact is negligible [29,43]. This is also the approach adopted for FRP in many guidelines [44,45] and for FRCM [46,47], neglecting in any cases the matrix contribution.

#### 2.2.2. Bilinear Behavior

The bilinear behavior was modeled through two linear relationships (Figure 2). This simplified behavior is representative of the typical FRCM systems with synthetic fibers: basalt, glass, carbon, or PBO (Poliparafenilenbenzobisoxazolo) and high performance matrix [30,48]. The first line ends with the attainment of the first cracking in tension of the mortar matrix, *σ_cr_*, with a slope equal to the modulus of elasticity homogenized with respect to the dry fiber. In fact, please note that the system is composed of two (parallel) materials, hence the behavior is homogenized with respect to dry fiber thickness. Therefore, in the first slope, even if dominated by the matrix, the modulus of elasticity is higher than the modulus of elasticity of the matrix due to this homogenization. The analytical relationship used to determine the modulus of elasticity is as follows:

(5) $$E_{1}=\frac{E_{m}\cdot t_{m}+E_{f}\cdot t_{f}}{t_{f}},$$

in which:

*E_m_* is the modulus of elasticity of the mortar matrix;
*t_m_* is the thickness of the matrix layer;
*t_f_* is the equivalent thickness of the composite fiber.

In this first phase, the composite has perfect matrix–fiber adhesion; therefore, it is necessary to indicate a stiffness representative of the entire composite, starting from the modulus of elasticity of the individual components and their respective thicknesses.

The second part of the relationship is characterized by a slope connecting the failure of the fibers and the first cracking in tension of the mortar matrix, therefore:

(6) $$E_{2}=\frac{\sigma_{um}-\sigma_{cr}}{\varepsilon_{um}-\varepsilon_{cr}},$$

in which:

$\varepsilon_{cr}$ is the first cracking strain in tension of the matrix ($\varepsilon_{cr}=-f_{tm}/E_{m}$);
$\sigma_{cr}$ is the stress corresponding to $\varepsilon_{cr}$;
*f_tm_* is the tensile strength of the mortar matrix.

#### 2.2.3. Trilinear Behavior

The trilinear behavior is modeled by means of three linear relationships. Compared to the bilinear, it accounts for an imperfect matrix contribution, after cracking, with a reduced tension stiffening effect (Figure 2). It is sometimes found in systems with natural fibers [49,50]. The first linear behavior is similar to the bilinear model, however, after cracking, there is a noticeable increase in deformation, caused by the development of the cracks without a clear increase of stress, and in particular the second stage is modeled as a perfectly horizontal (zero stiffness) plateau in this study. Hence, the slope of the second line is zero and the transition strain, $\varepsilon_{2}$, before the third linear part is:

(7) $$\varepsilon_{2}=\frac{\sigma_{cr}}{E_{f}},$$

The third line has the same slope of the dry fibers and continues until the fiber failure in tension is reached (or a bond failure), with a slope *E*_3_ equal to *E_f_*, which corresponds to the modulus of elasticity of the dry fiber only. In fact, it is assumed that in this phase the only element of the composite to provide a stress contribution is the fiber fraction.

## 3. Structural Evaluation

### 3.1. Basic Assumptions

The knowledge of the bending moment and ultimate curvature capacity is key for strengthened cross sections where an excessive amount of reinforcement can be detrimental, in particular with regard to ductility and failure mode [19]. In the presence of materials with poor tensile strength, it is desirable to carry out the calculation without accounting for tensile strength [10].

The numerical analysis of the section is carried out on the basis of some simplified assumptions:

- Conservation of plain sections (classical assumption in the technical calculation of the cross sections, also known as the Bernoulli-Navier assumption);
- Validity of the Bernoulli principle—the shear deformability is neglected;
- The stress–strain relationship of the materials are known a priori;
- The stresses are dependent on the strain of the element alone, so that the effects of viscosity and shrinkage over time are neglected;
- It is assumed that the application of the load and the consequent deformation take place in a monotonous way, therefore not explicitly considering the behavior under cyclic loads;
- It assumes the perfect bond between masonry and reinforcement (without slip);
- The ultimate condition of the cross section is reached, either by crushing of the compressed masonry, or tensile failure of the composite system. With reference to the latter, it is assumed that the failure may occur due to the traction of the fiber or abrupt debonding of the composite;
- Composites are effective only in traction and never in compression (assumption due to composite slenderness).

### 3.2. P-M Domains

**Figure 3:**
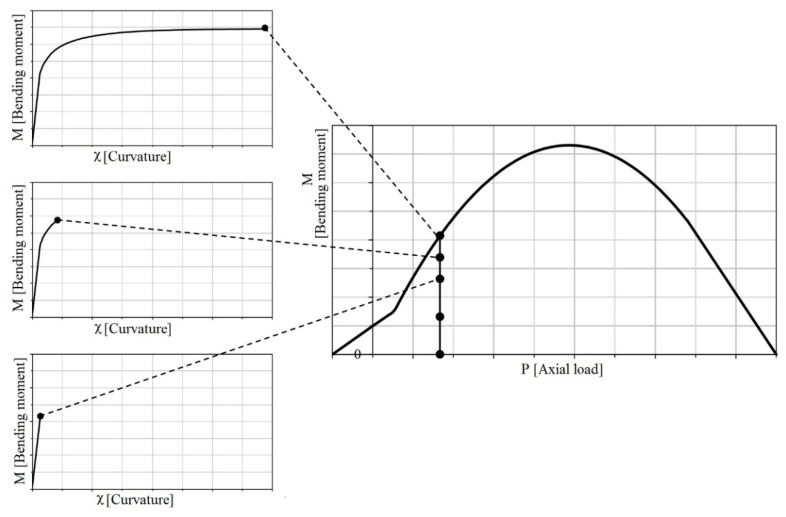
Parallelism between P-M domain and moment-curvature diagrams.

The construction of the P-M domain was carried out starting from the bending moment versus curvature diagrams, identifying the maximum values of bending moments at different axial loads (Figure 3). In a generic P-M domain, once the axial load is fixed, the points along a vertical line represent different steps at increasing curvature in the plane M-χ. In the P-M domain is reported the bending capacity for a given axial load, as the end point of the M-χ diagram, or the maximum value in general of the bending moment versus curvature diagrams.

### 3.3. Normalization of Results

The normalization allows one to provide generalizable results of whatever are the geometric and mechanical parameters, and therefore the outcomes are applicable to any masonry structural context. The proposed formulation is partly adapted from Reinforced Concrete (RC) cross sections. The main difference here is that mechanical fiber reinforcement ratio is usually lower and the stress–strain relationship is usually multilinear depending on the stress–strain relationship of composite with usually different hardening ratios, without any contribution in compression. Given a generic cross section, the following normalized parameters are introduced:

(8) $$p=\frac{P}{B\cdot H\cdot f_{c}}\;\;\;\;\;m=\frac{6\cdot M}{B\cdot H^{2}\cdot f_{c}},$$

in which:

*B* and *H* are respectively the base and height of the cross section;
*f_c_* is the compressive strength of the masonry;
*P* is the axial load;
*M* is the bending moment.

Looking at the trilinear behavior, normalization was performed with reference to the ultimate tensile strength of the dry fiber. In the assumption of perfect bond, it is possible to state:

(9) $$\varepsilon_{m}=\varepsilon_{f}=\varepsilon \;\;\;\to \;\;\;\frac{\sigma_{m}}{E_{m}}=\frac{\sigma_{f}}{E_{f}},$$

where

*ε_m_* is the strain of the mortar matrix;
*ε_f_* is the strain of the composite system (and of fibers) neglecting the thickness of the matrix.

By considering dry fibers, the following expression is valid:

(10) $$\sigma =\frac{\sigma_{m}\cdot A_{m}+\sigma_{f}\cdot A_{f}}{A_{f}}=\varepsilon \cdot E_{m}\cdot \rho_{m}+\varepsilon \cdot E_{f},$$

where *ρ_m_* is the ratio between the mortar matrix and the fiber cross section, hence fiber ratio. In this way it is possible to obtain the homogenized modulus of elasticity E_1_ as the slope of the first linear phase, which ends with the attainment of the cracking strain:

(11) $$E_{1}=\frac{\sigma }{\varepsilon }=E_{m}\cdot \rho_{m}+E_{f}\;\;\;\to \;\;\;\bar{E_{1}}=\frac{E_{1}}{f_{f}}=\frac{E_{m}\cdot \rho_{m}+E_{f}}{f_{f}}.$$

where *f_f_* is the tensile strength of the dry fiber.

The first portion ends with the crack strain of the mortar corresponding to a normalized stress, which can be evaluated as follows:

(12) $$\bar{\sigma_{cr}}=\varepsilon_{cr}\cdot \bar{E_{1}}=\frac{\varepsilon_{cr}\cdot \left(E_{m}\cdot \rho_{m}+E_{f}\right)}{f_{f}}=\frac{f_{tm}\cdot \left(E_{m}\cdot \rho_{m}+E_{f}\right)}{E_{m}\cdot f_{f}}=\frac{f_{tm}}{f_{f}}\left(\rho_{m}+\frac{E_{f}}{E_{m}}\right).$$

The slope of the third line is given simply by dividing the modulus of elasticity of the dry fiber *E_f_* by the fiber strength:

(13) $$\bar{E_{f}}=\frac{E_{f}}{f_{f}}.$$

All the previous quantities are dimensionless; please note that strain is already dimensionless. According to these assumptions, the stress–strain relationship can be expressed in normalized form:

(14) $$\bar{\sigma_{f}}=\{\begin{array}{c} \varepsilon \cdot \bar{E_{1}},\;\;\varepsilon_{cr}\leq \varepsilon \leq 0\\ \bar{\sigma_{cr}},\;\;\varepsilon_{2}<\varepsilon <\varepsilon_{cr}\\ \varepsilon \cdot \bar{E_{f}},\;\;\varepsilon \leq \varepsilon_{2} \end{array}.$$

As done for the trilinear behavior, the same can be repeated for bilinear behavior; the normalization is the same up to the point of cracking. The second linear part, whose slope is indicated with E_2_, should be divided by the dry fiber strength:

(15) $${\bar{E}}_{2}=\frac{E_{2}}{f_{f}}=(\frac{\sigma_{f}-\sigma_{cr}}{\varepsilon_{uf}-\varepsilon_{cr}})\cdot \frac{1}{f_{f}}.$$

And the stress–strain relationship can be expressed in normalized form

(16) $$\bar{\sigma_{f}}=\{\begin{array}{c} \varepsilon \cdot \bar{E_{1}},\;\;\varepsilon_{cr}\leq \varepsilon \leq 0\\ \varepsilon \cdot \bar{E_{2}},\;\;\varepsilon <\varepsilon_{cr} \end{array}.$$

Finally, the same can be repeated for the linear behavior, where the normalized modulus of elasticity of Equation (12) is used.

Hence, it has been demonstrated that every composite system can be expressed by means of a few normalized parameters: $\bar{E_{1}}$, $\bar{E_{2}}$, $\bar{E_{f}}$, $\bar{\sigma_{cr}}$, $\bar{\sigma_{f}}$ and $\rho_{m}$.

Similarly, it can be easily checked that the equilibrium equations leading to the moment curvature or the P-M domains can be similarly provided in normalized form, depending only on few normalized parameters: ω, and $\bar{\sigma_{f}}$ depending on the structure, and ψ, λ, ξ, related to model calculations [51], as the following discussed.

**Figure 4:**
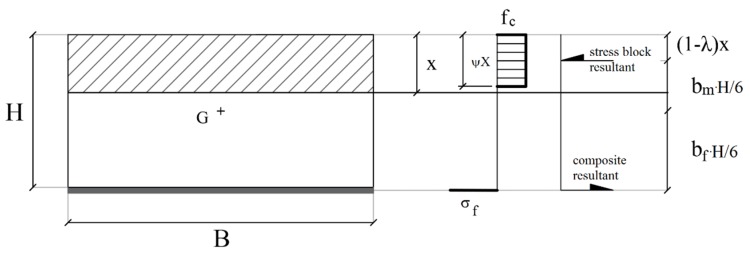
Equilibrium of the strengthened masonry cross section under no-tensile strength assumption of masonry.

The depth of the neutral axis, x, can be normalized with respect to cross section height, *ξ* = x/H, and the horizontal equilibrium equation is:

(17) $$p=\psi \cdot \xi +\omega \cdot \bar{\sigma_{f}}.$$

where:

*ψ* is the factor (dimensionless value) that correlate the real nonlinear stress distribution with the stress block resultant [51] (function of the maximum masonry strain), as shown in Figure 4:

Here, ω is the mechanical fiber reinforcement ratio defined as:

(18) $$\omega =\frac{A_{f}\cdot f_{f}}{B\cdot H\cdot f_{m}}.$$

The bending capacity is evaluated according to rotational equilibrium around the centroid of the cross section:

(19) $$m=\psi \cdot \xi \cdot b_{m}-\omega \cdot \bar{\sigma_{f}}\cdot b_{f}\;\;\;\to \;\;\;m=-6\left(1-\lambda \right)\psi \xi^{2}+6\psi \xi -3p.$$

where:

*b_m_* is the lever arm of masonry resultant normalized to H/6:

(20) $$b_{m}=-6\left(1-\lambda \right)\xi +3;$$

*b_f_* is the lever arm of FRCM resultant (i.e. H/2) normalized to H/6, hence it is equal to 3;

*λ* is the factor (dimensionless value) that correlates the real distance of the centroid of nonlinear stress distribution with the neutral axis depth [51] (function of the maximum concrete strain). Hence, it is confirmed that Equations (17) and (19) are functions only of the outlined normalized parameters.

## 4. Sensitivity Analysis

Sensitivity analysis was carried out considering appropriate values for the normalized stress–strain relationships of masonry and composite systems. The obtained results are dimensionless, hence, without regard to the geometrical and mechanical parameters of the cross section and of the composite system. The main objective was to analyze the mechanical performance at the variation of the applied mechanical percentages of strengthening, and how, with that strengthening amount, the cross section’s behavior changes according to the stress–strain behavior of the composite.

**Figure 5:**
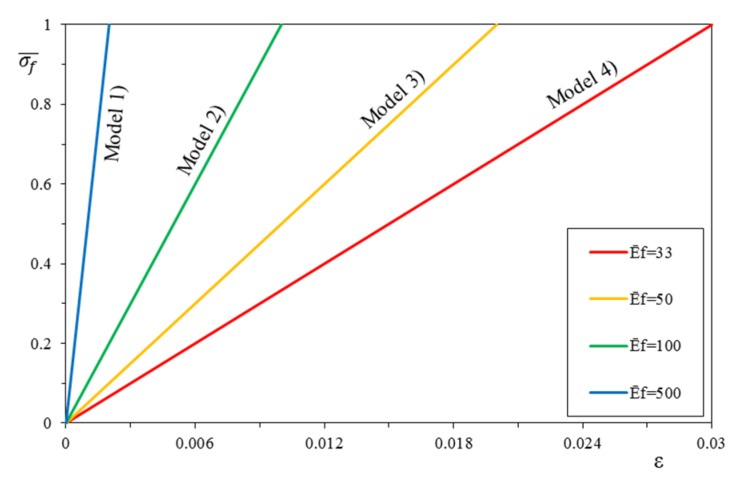
Linear stress–strain constitutive relationships (and corresponding *Models*).

The choice of the mechanical and geometrical parameters for the sensitivity analysis was carried out by analyzing a large amount of dry fibers and commercially available structural mortars in order to identify the typical mechanical characteristics and ranges. Please note that for FRCM there is the inorganic matrix, while for the FRP there is only dry fiber properties, and the matrix is neglected (i.e., only linear behavior). Then, in Table 1, the ranges of the geometrical and mechanical parameters are reported with regard to the composite systems and masonry. From the combination of the different extreme values of the intervals, a possible range of variability of the composite systems has been identified (the different stress–strain relationships have been called *models*). The normalized stiffness of the dry fiber ranged from $\bar{E_{f}}$ = 33 up to $\bar{E_{f}}$ = 500, generating four linear diagrams (${\bar{E}}_{f,model\;1}$ = 33, ${\bar{E}}_{f,model\;2}$ = 50, ${\bar{E}}_{f,model\;3}$ = 100, and ${\bar{E}}_{f,model\;4}$ = 500), as shown in Figure 5.

**Figure 6:**
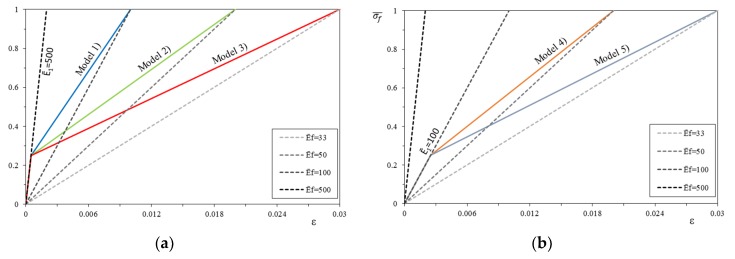

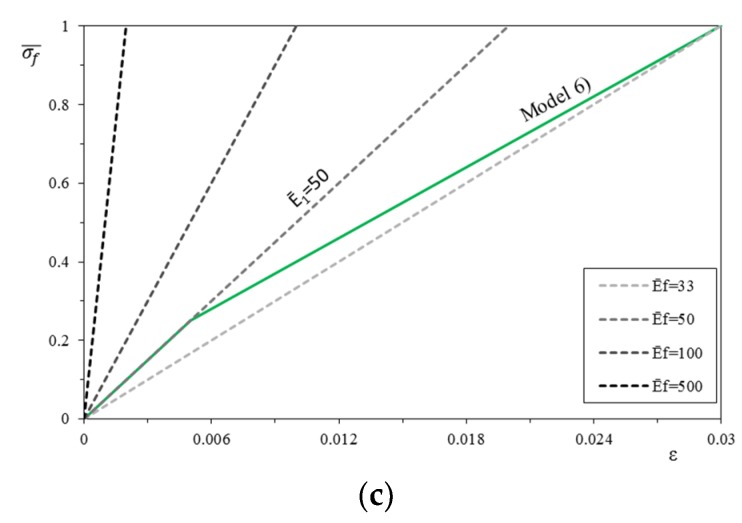
Bilinear stress–strain constitutive relationships (shown for $\bar{\sigma_{cr}}$ = 0.25 and corresponding *Models*): (**a**) Models 1, 2 and 3, (**b**) Models 4 and 5, (**c**) Model 6.

On the basis of the four linear constitutive relationships, twelve bilinear relationships (or *models*) were then constructed, in groups of 6, by changing the value of the first cracking stress of the mortar. In this case the change of the normalized slope of the first linear phase $\bar{E_{1}}$ has been taken at a normalized stress, $\bar{\sigma_{cr}}$, of 0.25 and 0.5 (*models* are plotted in solid line in Figure 6). The first three models (model 1, model 2, and model 3) present the same uncracked modulus of elasticity $\bar{E_{1}}=500$ but change the ultimate strain of the post-cracking modulus $\bar{E_{2}}$ calculated according to Equations (6) and (15). Models 4 and 5 have the uncracked modulus of elasticity $\bar{E_{1}}=100$ and the post-cracking modulus $\bar{E_{2}}$ is evaluated with the same approach. Model 6 has the uncracked modulus of elasticity $\bar{E_{1}}=50$ and the stiffness $\bar{E_{2}}$ is evaluated as previously discussed.

**Figure 7:**
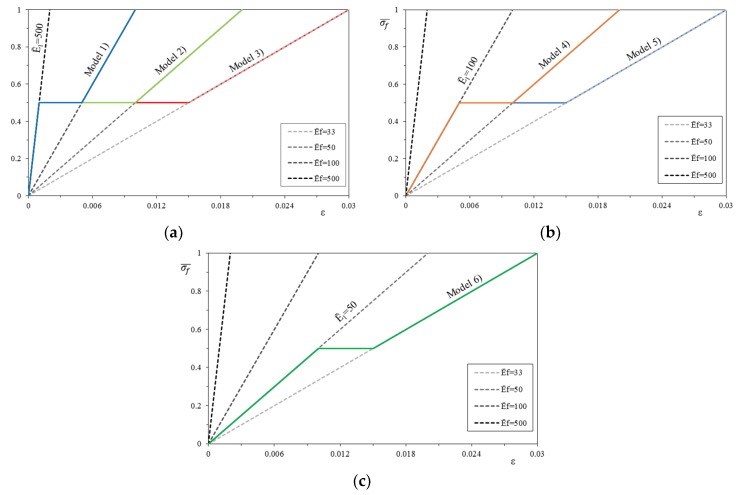
Trilinear stress–strain constitutive relationships (shown for $\bar{\sigma_{cr}}$ = 0.5 and corresponding *Models*): (**a**) Models 1, 2 and 3, (**b**) Models 4 and 5, (**c**) Model 6.

With the same approach of the bilinear constitutive relationships, it is possible to build as many trilinear stress–strain constitutive relationships (or *models*), always following the four basic linear relationships. The same variability of the tensile strength and stiffness of the mortar matrix is maintained, and a weak transition to the dry fiber behavior is assumed with a horizontal line, intersecting the different dry fiber behaviors (each *model* is in solid line in Figure 7). The first three *models* (model 1, model 2, and model 3) present both the same uncracked modulus $\bar{E_{1}}$ = 500 and the normalized first cracking strength $\bar{\sigma_{cr}}$ (i.e., the corresponding strain $\varepsilon_{cr}$), but the strain corresponding to the stress $\sigma_{cr}$ at the end of the constant stress–strain line $\varepsilon_{2}$ changes. It is evaluated as a function of the normalized modulus of elasticity of the dry fiber $\bar{E_{f}}$, respectively, equal to 100, 50, and 33. Models 4 and 5 are obtained with the same approach, fixing the uncracked modulus $\bar{E_{1}}$ = 100 and changing the modulus of the dry fiber $\bar{E_{f}}$, respectively, equal to 50 and 33. Model 6 has the uncracked modulus $\bar{E_{1}}$ = 50 and the dry fiber $\bar{E_{f}}$ is equal to 33.

For each of the previously defined stress–strain constitutive relationships, the bending capacity of the strengthened masonry section was evaluated in terms of the *p-m* interaction domain.

### 4.1. Linear Behavior

**Figure 8:**
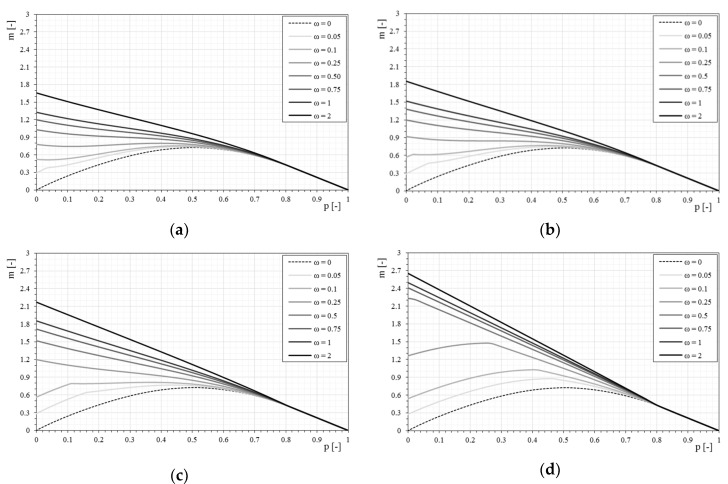
The *p-m* interaction domains with the variation of the stress–strain constitutive relationships of the reinforcement: (**a**) Model 1-$\bar{E_{f}}$ = 33; (**b**) Model 2-$\bar{E_{f}}$ = 50; (**c**) Model 3-$\bar{E_{f}}$ = 100; (**d**) Model 4-$\bar{E_{f}}$ = 500.

Figure 8 show the interaction domains in the case of linear stress–strain constitutive relationships of the reinforcement when the dimensionless parameter $\bar{E_{f}}$ changes and the mechanical fiber reinforcement ratio, ω, also changes.

The influence of the mechanical fiber reinforcement ratio is higher as the stiffness of the fiber increases. The influence of the reinforcement tends to reduce with the increase of compression on the section, as clearly seen in Figure 8. This assumption is due to the basic assumptions adopted, and in particular because the strengthening system is not reactive to compression.

**Figure 9:**
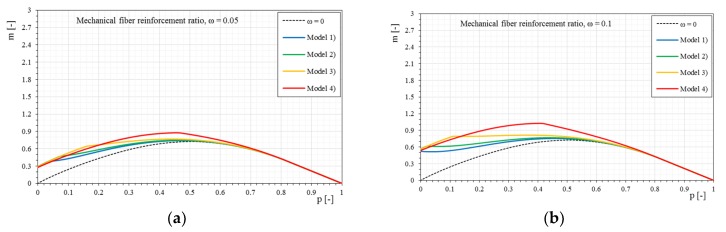

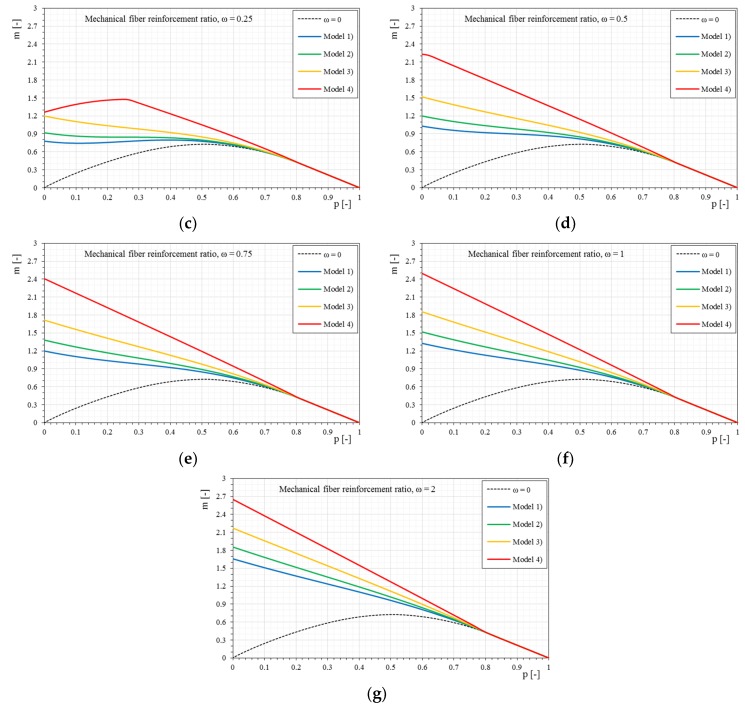
The *p-m* interaction domains with linear stress–strain constitutive relationships of the reinforcement as the mechanical fiber reinforcement ratio, ω, changes: (**a**) ω = 0.05, (**b**) ω = 0.1, (**c**) ω = 0.25, (**d**) ω = 0.5, (**e**) ω = 0.75, (**f**) ω = 1, (**g**) ω = 2.

Once the interaction domains have been defined when the mechanical ratio ω changes, it is interesting to observe how the domain varies for a given mechanical fiber reinforcement ratio ω, only varying the constitutive relationships of the applied reinforcement (dimensionless parameter $\bar{E_{f}}$), as shown in Figure 9.

For all the cases it is observed how, increasing the stiffness of the reinforcement (dimensionless parameter $\bar{E_{f}}$), the interaction domains tend to expand. However, it is interesting to note that for low mechanical fiber reinforcement ratios the influence of the reinforcement stiffness alone does not generate a proportional increase of the interaction domain but it is strongly altered also in the shape. As the fiber reinforcement ratio increases, the interaction domains have more homogeneous increases as seen in the previous figures. In the same way, the interaction domains are proposed in the following, based on the bilinear stress–strain constitutive relationships.

### 4.2. Bilinear Behavior

**Figure 10:**
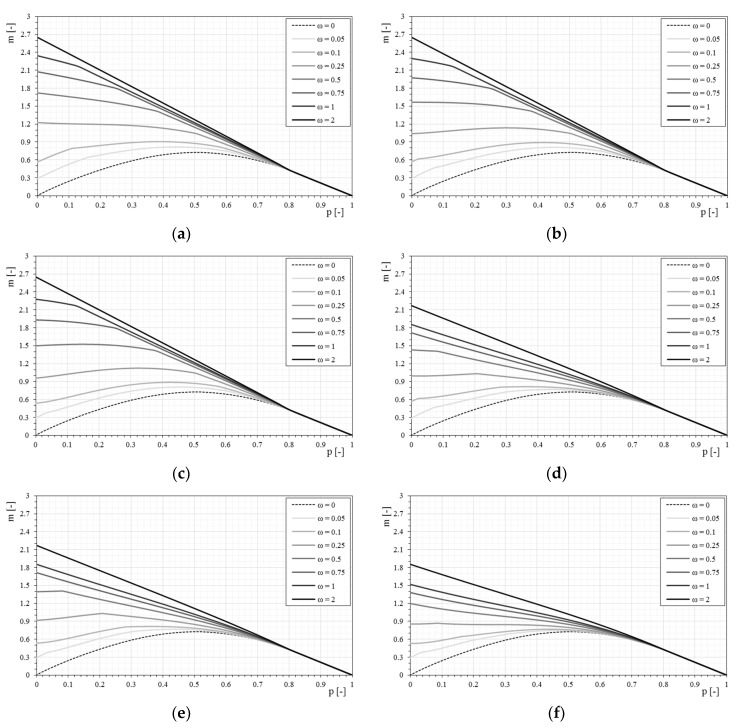
The *p-m* interaction domains with bilinear stress–strain constitutive relationships of the reinforcement ($\bar{\sigma_{cr}}$ = 0.5): (**a**) Model 1-$\bar{E_{1}}$ = 500; (**b**) Model 2-$\bar{E_{1}}$ = 500; (**c**) Model 3-$\bar{E_{1}}$ = 500; (**d**) Model 4-$\bar{E_{1}}$ = 100; (**e**) Model 5-$\bar{E_{1}}$ = 100; (**f**) Model 6-$\bar{E_{1}}$ = 50.

Figure 10 show the *p-m* interaction domains in the case of a bilinear stress–strain constitutive relationship of the reinforcement and a normalized tensile strength of the matrix equal to 0.5, as the mechanical fiber reinforcement ratio varies. The use of a bilinear relationship modifies the *p-m* interaction domains for low values of axial load. In these conditions, the failure of the section is mainly due to the compression of the masonry, while the reinforcement remains in the elastic field. Under these conditions, the reinforcement can rely on the behavior of the composite fiber plus the matrix, and not only of the fiber component. The dependence of the interaction domain from the stress–strain constitutive relationship is strictly related to the stiffness of the dimensionless parameters $\bar{E_{1}}$ (matrix stiffness) and $\bar{E_{f}}$ (stiffness of the reinforcement). As the axial load increases, the interaction domain is less affected by the type of constitutive relationship, as seen in Figure 10.

**Figure 11:**
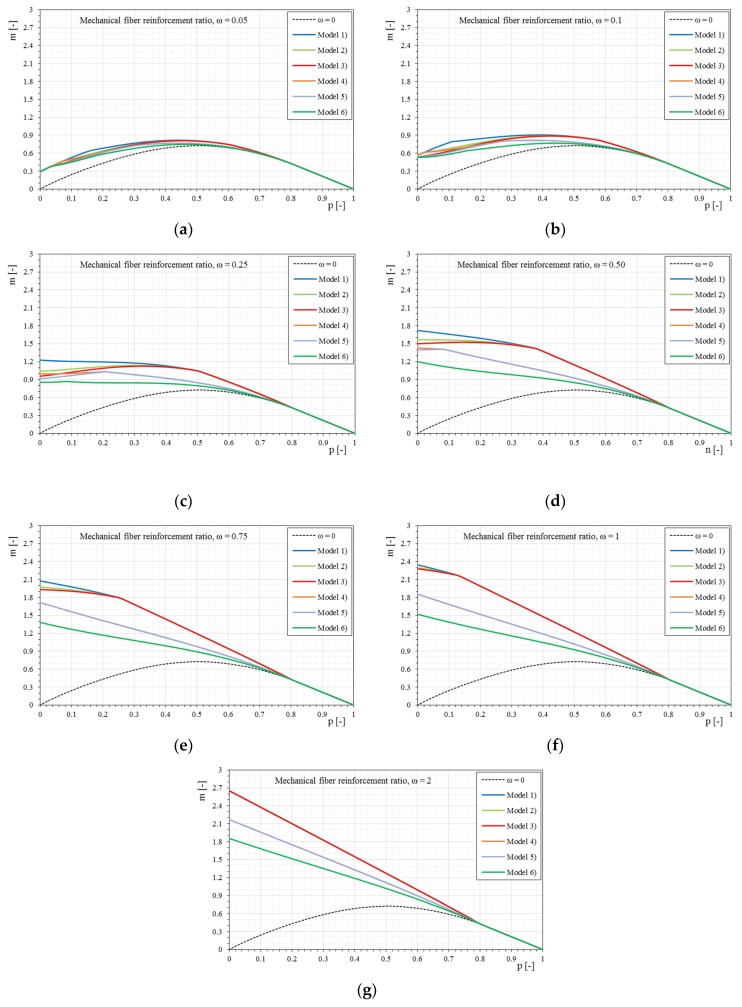
The *p-m* interaction domain with bilinear stress–strain constitutive relationships of the reinforcement ($\bar{\sigma_{cr}}$ = 0.5) as the mechanical fiber reinforcement ratio, ω, changes. (**a**) ω = 0.05, (**b**) ω = 0.1, (**c**) ω = 0.25, (**d**) ω = 0.5, (**e**) ω = 0.75, (**f**) ω = 1, (**g**) ω = 2.

In Figure 11 the response of the strengthened masonry section to the variation of the bilinear constitutive relationship by merging the *p-m* domains for the same mechanical fiber reinforcement ratio is analyzed. The effect of the bilinear constitutive relationship is, as previously mentioned, strongly visible for low normal stress values and increases as the mechanical fiber reinforcement ratio increases.

With the bilinear constitutive relationships, at the same time, the matrix (mortar) is also taken into account, while the simply linear relationships neglect this contribution. Thus, when changing the tensile strength of the mortar, the constitutive relationship of the reinforcement necessarily varies. Furthermore, some curves can almost overlap because they share the uncracked modulus of elasticity $\bar{E_{1}}$, and if failure occurs with reinforcement in the first linear range, the masonry behaves equally.

**Figure 12:**
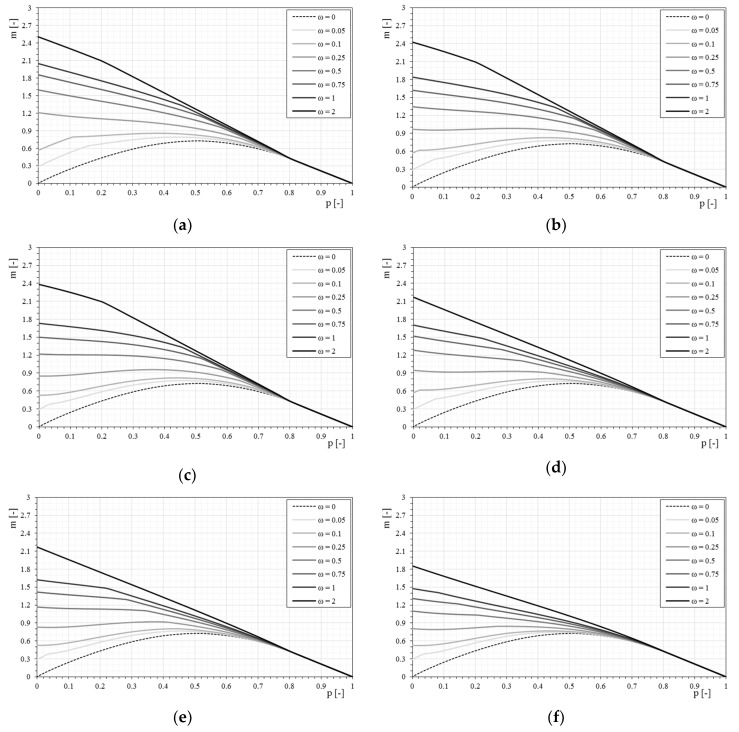
The *p-m* interaction domain with bilinear stress–strain constitutive relationships of the reinforcement ($\bar{\sigma_{cr}}$ = 0.25): (**a**) Model 1-$\bar{E_{1}}$ = 500; (**b**) Model 2-$\bar{E_{1}}$ = 500; (**c**) Model 3-$\bar{E_{1}}$ = 500; (**d**) Model 4-$\bar{E_{1}}$ = 100; (**e**) Model 5-$\bar{E_{1}}$ = 100; (**f**) Model 6-$\bar{E_{1}}$ = 50.

It will be shown (in Figure 12) how the response of the section changes with the same characteristics of the fibers by simply modifying the tensile strength of the mortar, bringing the dimensionless cracking stress from 0.5 to 0.25.

The influence of the constitutive relationship, in particular of the matrix, is observed for even lower values of axial stress while the tensile strength of the matrix decreases. In fact, when the tensile strength of the matrix is reduced, the bilinear relationship tends to provide results that are more similar to the linear relationship of the fiber alone.

**Figure 13:**
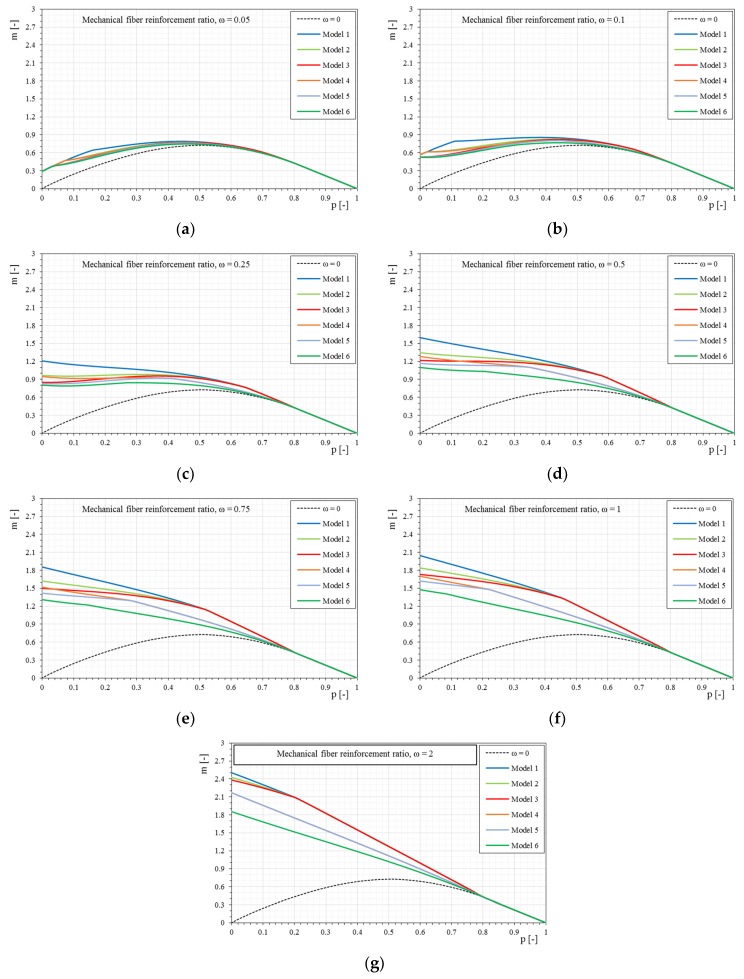
The *p-m* interaction domains with bilinear stress–strain constitutive relationships of the reinforcement ($\bar{\sigma_{cr}}$ = 0.25) as the mechanical fiber reinforcement ratio, ω, changes. (**a**) ω = 0.05, (**b**) ω = 0.1, (**c**) ω = 0.25, (**d**) ω = 0.5, (**e**) ω = 0.75, (**f**) ω = 1, (**g**) ω = 2.

The influence of the tensile strength of the matrix is also visible once fixed for the mechanical fiber reinforcement ratio of the reinforcement and varying the characteristics of the bilinear stress–strain constitutive relationships (Figure 13). With respect to the case of the linear relationship, with the reduction of the tensile strength of the matrix, the bilinear stress–strain constitutive relationship influences the *p-m* domain less markedly.

### 4.3. Trilinear Behavior

**Figure 14:**
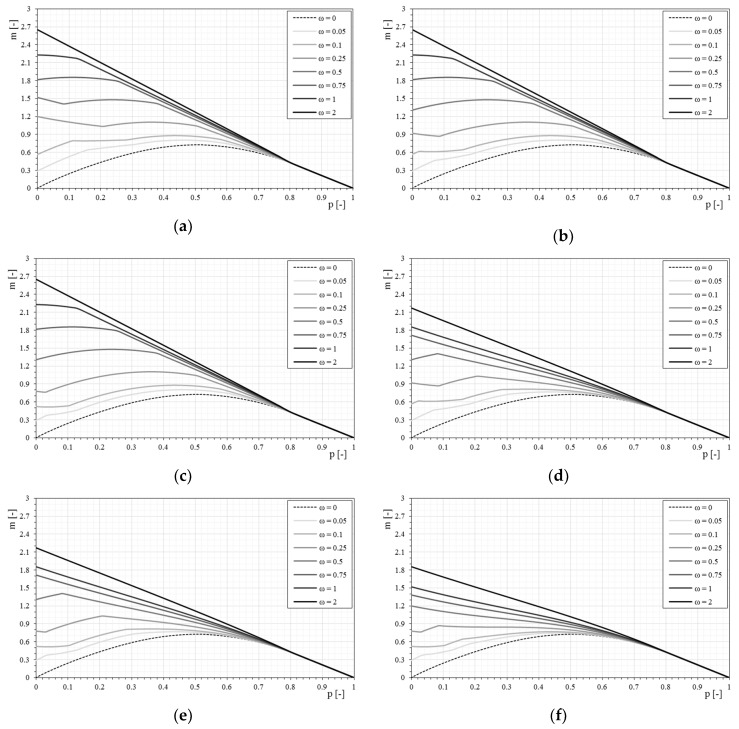
The *p-m* interaction domain with trilinear stress–strain constitutive relationships of the reinforcement ($\bar{\sigma_{cr}}$ = 0.5): (**a**) Model 1; (**b**) Model 2; (**c**) Model 3; (**d**) Model 4; (**e**) Model 5; (**f**) Model 6.

Figure 14 show the *p-m* interaction domains in the case of a trilinear stress–strain constitutive relationship of the reinforcement and a normalized tensile strength of the matrix equal to 0.5, as the mechanical fiber reinforcement ratio varies. In this case too, the effect of the trilinear stress–strain constitutive relationship is clear at low axial load values, and with greater fiber ratios it is even more evident. However, it is noted that for high mechanical fiber ratios, the domains calculated with bilinear and trilinear relationships tend to overlap. This effect is essentially due to the stress level of the reinforcement, which is reduced with an increase of the mechanical fiber reinforcement ratio. Under these conditions the bilinear and trilinear relationships tend to coincide. The transition from the bilinear to the trilinear relationship is always visible, however, as the initial stiffness $\bar{E_{1}}$ decreases, the three relationships tend to coincide, especially for high values of mechanical fiber ratios.

**Figure 15:**
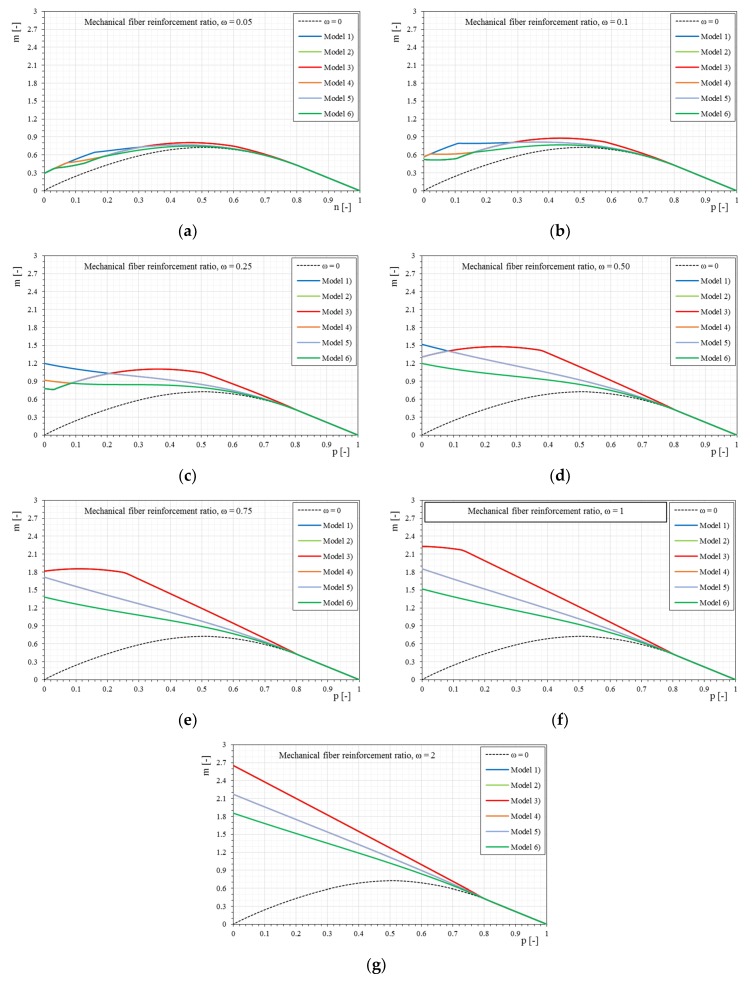
The *p-m* interaction domains with trilinear stress–strain constitutive relationships of the reinforcement ($\bar{\sigma_{cr}}$ = 0.5) as the mechanical fiber reinforcement ratio, ω, changes. (**a**) ω = 0.05, (**b**) ω = 0.1, (**c**) ω = 0.25, (**d**) ω = 0.5, € ω = 0.75, (**f**) ω = 1, (**g**) ω = 2.

Figure 15 shows the comparison of the *p-m* interaction domains with the variation of the trilinear stress–strain constitutive relationships, maintaining the same mechanical fiber ratio of reinforcement. It is interesting to note, once again, that as the mechanical fiber ratio increases, the interaction domains are not essentially dependent on the initial stiffness of the composite, given that the stress level decreases as the mechanical percentage increases. Furthermore, some curves can be almost overlapped, as in the bilinear case, because they share the uncracked modulus of elasticity $\bar{E_{1}}$ or another part of the relationship.

### 4.4. Different Stress–Strain Constitutive Relationships

**Figure 16:**
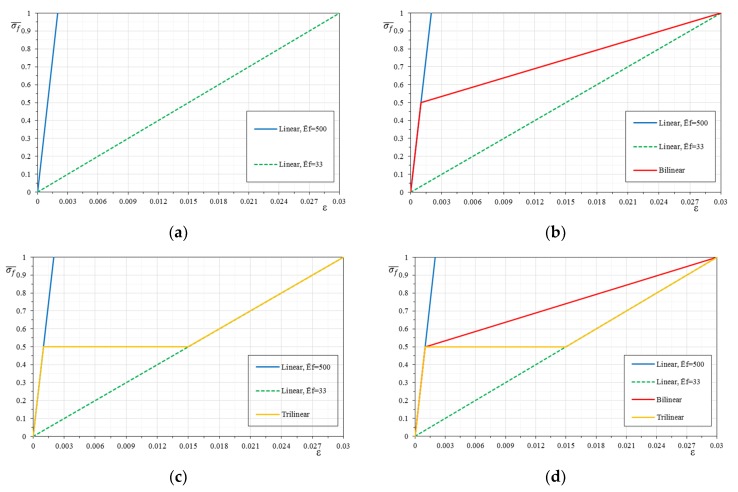
Comparisons of linear-bilinear-trilinear stress–strain constitutive relationships of the reinforcement: (**a**) definition of the linear stress–strain constitutive relationships - $\bar{E_{f}}$ = 33 and $\bar{E_{f}}$ = 500, (**b**) definition of the bilinear stress–strain constitutive relationship, (**c**) definition of the trilinear stress–strain constitutive relationship, (**d**) stress-strain relationships comparison.

**Figure 17:**
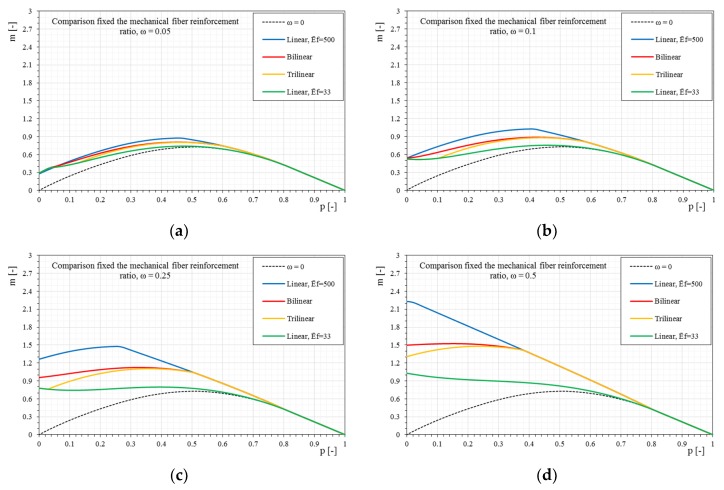

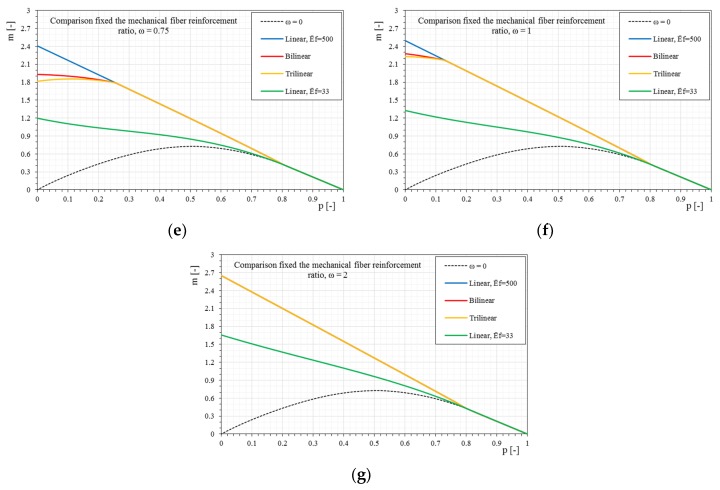
The *p-m* interaction domains as a function of the linear-bilinear-trilinear stress–strain constitutive relationships of the reinforcement and as the mechanical fiber reinforcement ratio, ω, changes. (**a**) ω = 0.05, (**b**) ω = 0.1, (**c**) ω = 0.25, (**d**) ω = 0.5, € ω = 0.75, (**f**) ω = 1, (**g**) ω = 2.

Figure 16 and Figure 17 show a direct comparison of different stress–strain constitutive relationships and mechanical fiber reinforcement ratios. It is interesting to note the influence of the stress–strain constitutive relationships (linear, bilinear, and trilinear) as the mechanical fiber reinforcement ratio varies. Therefore, there are interaction domains assuming the same type of reinforcement but modeled with the three different relationships (Figure 16).

The comparison of the *p-m* interaction domains shows a clear result, as previously observed, but in this case it is undoubtedly more evident. In particular, once a mechanical fiber reinforcement ratio has been exceeded, for the case shown where ω = 1, there is no longer a distinction between the interaction domains as the constitutive relationships of the reinforcement change. In particular, once the composite is modeled according to a bilinear or trilinear relationship, the interaction domain has negligible variations. It is observed how, while increasing the mechanical fiber reinforcement ratio, the interaction domains tend to overlap completely. This is fundamentally linked to the high fiber reinforcement ratio applied. In fact, with reference to such high amounts of reinforcements, the stress of the composite hardly exceeds the elastic phase, and since the bilinear and trilinear constitutive relationships share the same initial elastic behavior, the overlapping of the interaction domains is reasonable. In the case of different initial slopes, the influence of the constitutive relationship on the ultimate behavior of the strengthened masonry always tends to be less evident as the mechanical fiber reinforcement ratio of the reinforcement increases, just because the stress of the reinforcement at ultimate conditions reduces.

## 5. Conclusions

In the present work the behavior of masonry sections strengthened with FRP or FRCM composite systems was evaluated when varying the different mechanical parameters. The variability of the behavior has been analyzed, changing the mechanical characteristics of the reinforcement and the type of constitutive relationships. All results were generalized through an adimensionalization process, providing extremely useful results for the design phase. Several *p-m* interaction domains were generated, changing not only the constitutive relationship of the reinforcement, but also the fiber reinforcement ratio.

It has been interesting to observe how the section exploited the same behavior, no longer depending on the type of constitutive relationship used for the composite once a certain mechanical fiber reinforcement ratio of reinforcement is exceeded. In fact, for sufficiently high amounts of reinforcement, the stress of the composite hardly exceeds the elastic branch, and since the bilinear and trilinear constitutive relationships share the same initial part of the linear branch, the overlapping of the *p-m* interaction domains is justified.

In practical applications, in the design of interventions, more prescriptive than performance-based approaches generally lead to an overestimation of the effective amount of reinforcement required. The proposed theory contributes to the implementation of performance-based design. The type of reinforcement (i.e., design of the constitutive model) can be considered in order to optimize the structural behavior of the strengthened masonry. The proposed theory allows assessment of both the flexural capacity and the ductility capacity, as a future development. This represents an important aspect in assessment and strengthening, especially of poor masonry, where higher mechanical fiber reinforcement ratios or unbalanced mechanical properties could promote brittle behavior. However, it has been shown in several scientific papers how often even very low amounts of reinforcement are sufficient to significantly increase the capacity of a masonry element. For low mechanical fiber reinforcement ratios, the type of constitutive relationship becomes a key aspect. In this case, the modeling of the reinforcement plays a fundamental role and the type of constitutive relationships to be adopted (linear, bi-linear, or tri-linear) is strongly correlated to the type of reinforcement chosen, e.g., FRP versus FRCM. Therefore, the results provided in a dimensionless form are the basis for a valid support to the design of interventions using fiber composites on masonry structures.

## Figures and Tables

**Table 1:** Range of the parameters characterizing masonry, dry fibers, and mortar matrix.

Parameter	Minimum	Maximum
Masonry strength, *f_c_*	1 MPa	10 MPa
Modulus of elasticity of the masonry, *E_c_*	1 GPa	5 GPa
Dry fiber thickness, *t_f_*	0.1 mm	1 mm
Dry fiber strength, *f_f_*	500 MPa	3000 MPa
Modulus of elasticity of the fry fiber, *E_f_*	50 GPa	250 GPa
Mortar matrix thickness, *t_m_*	5 mm	20 mm
$\mathrm{Mortar}\;\mathrm{matrix}\;\mathrm{strength},\;f_{tm}$	0.5 MPa	20 MPa
Modulus of elasticity of the mortar matrix, *E_m_*	5 GPa	22 GPa

## Abbreviations

$\bar{E_{1}}$	normalized uncracked modulus of elasticity of the composite homogenized on the dry fiber
$\bar{E_{2}}$	normalized post-cracking modulus of the composite homogenized to the dry fiber
$\bar{E_{f}}$	normalized modulus of elasticity of the dry fiber
$\bar{\sigma_{cr}}$	normalized first cracking strength of the mortar matrix
$\bar{\sigma_{f}}$	stress of the composite normalized to the dry fiber
$A_{f}$	cross section of dry fiber
$A_{m}$	cross section of matrix
$E_{1}$	uncracked modulus of elasticity of the composite homogenized to the dry fiber
$E_{2}$	post- uncracked modulus of elasticity of the composite homogenized to the dry fiber
$\varepsilon_{2}$	strain corresponding to the stress $\sigma_{cr}$ at the end of constant stress-strain curve
$\varepsilon_{cr}$	strain corresponding to the stress $\sigma_{cr}$
$\varepsilon_{m}$	strain of the matrix (and masonry due to perfect bond assumption)
$\varepsilon_{um}$	ultimate strain of the composite
$\sigma_{um}$	ultimate stress of the fiber obtained from the bond tests
B	base of the cross section
b_f_	lever arm of FRCM resultant (i.e. H/2) normalized to H/6, hence it is equal to 3
b_m_	lever arm of masonry resultant normalized to H/6
E_c_	modulus of elasticity of the masonry
E_f_	modulus of elasticity of the dry fiber
E_m_	modulus of elasticity of the mortar matrix
f_c_	compressive strength of masonry
f_f_	tensile strength of the dry fiber
f_t_	tensile strength of masonry
f_tm_	tensile strength of mortar matrix
H	height of the cross section
m	normalized bending moment M
p	normalized axial load P
t_f_	equivalent thickness of the composite fiber
t_m_	thickness of the mortar layer
x	depth of the neutral axis
ε	generic strain of masonry or composite
ε_k_	conventional yielding strain of masonry in compression
ε_t_	conventional ultimate strain of masonry in tension
ε_u_	conventional ultimate strain of masonry in compression
λ	dimensionless factor that correlates the real distance of centroid of nonlinear stress distribution with the neutral axis depth
ξ	neutral axis normalized with respect to cross section height
ρ_m_	ratio of the fiber over matrix cross sections
σ_cr_	homogenized first cracking in tension of the mortar matrix
σ_m_	stress of the mortar matrix
ψ	dimensionless factor that correlates the real nonlinear stress distribution with the stress block resultant
ω	mechanical fiber reinforcement ratio
